# Quinolinate Phosphoribosyltransferase Promotes Invasiveness of Breast Cancer Through Myosin Light Chain Phosphorylation

**DOI:** 10.3389/fendo.2020.621944

**Published:** 2021-02-04

**Authors:** Chien-Liang Liu, Shih-Ping Cheng, Ming-Jen Chen, Chi-Hsin Lin, Shan-Na Chen, Yi-Hue Kuo, Yuan-Ching Chang

**Affiliations:** ^1^ Department of Surgery, MacKay Memorial Hospital, Taipei, Taiwan; ^2^ Department of Surgery, School of Medicine, Mackay Medical College, New Taipei City, Taiwan; ^3^ Department of Medical Research, MacKay Memorial Hospital, Taipei, Taiwan; ^4^ Department of Bioscience Technology, Chung Yuan Christian University, Taoyuan City, Taiwan

**Keywords:** quinolinate phosphoribosyltransferase, NAD, myosin light chain, neoplasm invasiveness, breast cancer

## Abstract

Perturbed Nicotinamide adenine dinucleotide (NAD^+^) homeostasis is involved in cancer progression and metastasis. Quinolinate phosphoribosyltransferase (QPRT) is the rate-limiting enzyme in the kynurenine pathway participating in NAD^+^ generation. In this study, we demonstrated that QPRT expression was upregulated in invasive breast cancer and spontaneous mammary tumors from MMTV-PyVT transgenic mice. Knockdown of QPRT expression inhibited breast cancer cell migration and invasion. Consistently, ectopic expression of QPRT promoted cell migration and invasion in breast cancer cells. Treatment with QPRT inhibitor (phthalic acid) or P2Y_11_ antagonist (NF340) could reverse the QPRT-induced invasiveness and phosphorylation of myosin light chain. Similar reversibility could be observed following treatment with Rho inhibitor (Y16), ROCK inhibitor (Y27632), PLC inhibitor (U73122), or MLCK inhibitor (ML7). Altogether, these results indicate that QPRT enhanced breast cancer invasiveness probably through purinergic signaling and might be a potential prognostic indicator and therapeutic target in breast cancer.

## Introduction

Breast cancer is the most common malignant disease among women and represents a major healthcare burden (1). The incidence of breast cancer has been increasing worldwide, including in Taiwan (2, 3). Despite tremendous advances in the diagnosis and treatment of breast cancer, an increase in breast cancer mortality rates without regional disparities has been observed (4). Developing new therapeutic strategies that target cancer progression and metastasis will be essential to improve patient outcomes.

Nicotinamide adenine dinucleotide (NAD^+^) and its reduced form NADH play an important role in biogenesis and redox balance of the human body (5). There is growing evidence of perturbed NAD^+^ homeostasis contributing to various disease states, including cancer and aging (6). In multiple cancer types, enzymes involving NAD^+^ metabolism are aberrantly expressed or dysregulated. One of the well-studied targets is nicotinamide phosphoribosyltransferase (NAMPT), the rate-limiting enzyme of the NAD^+^ salvage pathway (7). High NAMPT expression was associated with aggressive biological features in breast cancer and other malignancies (8, 9). Translational potential by genetic knockdown of NAMPT or pharmacologic inhibition has been rigorously evaluated in preclinical models.

In addition to the salvage pathway, NAD^+^ can be synthesized from a simple amino acid, tryptophan, *via* the *de novo* pathway (10). Quinolinate phosphoribosyltransferase (QPRT) is the final and rate-limiting enzyme in the kynurenine pathway (11). The role of QPRT in cancer has not been well studied. Higher QPRT expression was noted in aggressive glioblastomas than in low-grade gliomas (12). Recently, QPRT was identified as a crucial prognostic gene that was significantly associated with breast cancer overall survival (13). In this study, we aimed to explore the clinicopathological significance of QPRT expression in breast cancer and its potential biological mechanisms.

## Materials and Methods

### Cell Lines and Reagents

Human breast cancer cell lines (BT-20, T-47D, SK-BR-3, MCF-7, MDA-MB-468, MDA-MB-157, BT-474, DU4475, and MDA-MB-231) were all purchased from the American Type Culture Collection, Manassas, VA. An additional MCF-7 cell line derived from Dr. Jose Baselga’s laboratory (Memorial Sloan Kettering Cancer Center, New York, NY) was kindly provided by Dr. Yen-Shen Lu (National Taiwan University Hospital, Taipei, Taiwan) (14). Cell line authentication by short tandem repeat sequencing was performed to check for cross-contamination. BT-20 cells were grown in Eagle’s Minimum Essential Medium supplemented with 10% fetal bovine serum (FBS). MDA-MB-468 and MDA-MB-231 cells were grown in Leibovitz’s L-15 Medium supplemented with 10% FBS. All cells were maintained at 37°C in a 5% CO_2_ humidified atmosphere.

Cisplatin was purchased from Fresenius Kabi, Viman Nagar, India. Phthalic acid was obtained from Sigma-Aldrich, Merck KGaA, Darmstadt, Germany. For specific inhibitors, Y16 was obtained from MedChemExpress, Monmouth Junction, NJ; selisistat and olaparib from Selleck Chemicals, Houston, TX; NF340, Y27632, U73122, and ML7 from ApexBio Technology, Houston, TX.

### Public Databases and Bioinformatic Analysis

The clinicopathologic profile and mRNA expression data of The Cancer Genome Atlas (TCGA) breast cancer dataset were downloaded through the Genomic Data Commons Data Portal (https://portal.gdc.cancer.gov/) (15). High and low QPRT expression groups were assigned by the median split method, and overall survival between the groups was compared with the log-rank test statistic. Transcriptome data from tumor samples of the top 25 percent and the bottom 25 percent of QPRT expression were further examined, and gene set enrichment analysis (GSEA) was performed to explore the potential significance of differential QPRT expression (16). Additionally, we used a meta-analysis of databases, the KM Plotter Online Tool (https://kmplot.com/analysis/), to validate the relationship between QPRT expression and clinical outcomes (recurrence-free survival and distant metastasis-free survival) (17).

### Immunohistochemical (IHC) Staining

Tissue microarrays of breast neoplasms were obtained from Pantomics Inc., Fairfield, CA. The BB08015 set contained 48 tissue cores from 24 patients, and BC08118a contained 100 cores from 100 patients. Overall, 10 normal breast tissues, 20 cases of ductal carcinoma *in situ* (DCIS), and 94 cases of invasive carcinoma were included in the analysis. Mouse anti-human QPRT antibody was purchased from GeneTex, Irvine, CA. The tissue microarray slides were deparaffinized using xylene and rehydrated using serial gradient ethanol. The anti-QPRT antibody was diluted to 1:100. IHC staining was performed as we previously reported (18). Negative control slides were obtained by omitting the primary antibody incubation, and normal liver tissues were used as positive controls.

QPRT protein expression was quantified according to the intensity and extent of immunoreactivity. When the breast epithelial cells showed no positivity or <10% positive staining, they were scored as 0 and 1, respectively. When 10%–50% or >50% positive staining was observed, they were scored as 2 and 3, respectively. The IHC scores of two cores from the same patient were averaged.

### Transfection

Lentiviral plasmid vector pLKO.1-puro with short hairpin RNA (shRNA) specific for QPRT and lentiviral vector with control shRNA were purchased from Sigma-Aldrich. To knock down the QPRT expression, MDA-MB-468 and BT-20 cells were transduced with lentivirus in the presence of polybrene (Sigma-Aldrich) and selected with puromycin (InvivoGen, San Diego, CA). Knockdown efficacy was confirmed by real-time quantitative polymerase chain reaction and western blotting.

To overexpress QPRT in breast cancer cells, pCMV6-entry empty vector and pCMV6-entry QPRT expression constructs were purchased from OriGene Technologies, Rockville, MD. We transfected MDA-MB-231 cells with the constructs using Lipofectamine 3000 reagent (Thermo Fisher Scientific, Waltham, MA) according to the manufacturer’s protocol. QPRT overexpression was confirmed by western blotting 72 h after transfection.

### Cell Viability

Cell growth was evaluated in MDA-MB-468 and BT-20 cells stably transfected with a control shRNA or QPRT-targeting shRNA for 24 to 96 h. MDA-MB-231 cells transfected with pCMV6-entry or pCMV6-QPRT were treated with increasing doses (0.1, 1, 10, 100, and 1000 μM) of cisplatin for 24 or 48 h. Cell viability was determined by the CellTiter Aqueous One Solution Cell Proliferation (MTS) Assay (Promega, Madison, WI) as previously described (19).

### Migration and Invasion Assay

The migration and invasion assays were performed as described (20). Cells in serum-free medium were seeded onto the upper Transwell insert with 8-μm pores of polycarbonate membrane (Corning Life Sciences, Tewksbury, MA). For invasion assay, BioCoat cell culture inserts pre-coated with Matrigel matrix (Corning Life Sciences) were used. The lower chamber contained the complete culture medium. The cells migrated or invaded through the insert membrane were fixed and stained with Diff-Quick (Sysmex, Kobe, Japan). The numbers of migrated or invaded cells were counted under the microscope from five random fields.

### NAD^+^ Quantification

Intracellular NAD^+^ and NADH levels were measured using the NAD/NADH Assay Kit (ab65348; Abcam, Cambridge, UK) according to the manufacturer’s protocol. Briefly, cells were extracted with the NAD/NADH extraction buffer and filtered through a 10 kD spin column to remove enzymes that consume NADH. To detect the NADH only, decomposition was performed by heating the samples at 60°C for 30 min. As such, NAD^+^ was decomposed while the NADH was intact. The decomposition step was omitted in the detection of total NAD^+^ and NADH. NAD^+^ in the samples was then converted to NADH by adding NADH developer. Concentrations of NADH in the samples were derived from the standard curve. NAD^+^/NADH ratio was calculated as ((total NAD^+^ and NADH) - NADH)/NADH.

### Immunoblot

Proteins extracted from total cellular lysates were subjected to SDS-PAGE followed by transfer to polyvinylidene fluoride membranes (21). The membranes were incubated with the following primary antibodies: anti-QPRT (GTX83743; GeneTex), anti-phospho-ERK1/2 ^Thr202/Tyr204^ (#9101), anti-phospho-AKT ^Ser473^ (#9271), anti-phospho-GSK3β ^Ser9^ (#9336), anti-phospho-Smad2 ^Ser465/467^/Smad3 ^Ser423/425^ (#8828), anti-phospho-MLC2 ^Ser19^ (#3671), and MLC2 (#8505). All antibodies were obtained from Cell Signaling Technology, Danvers, MA unless otherwise specified. Anti-β-actin (A5441; Sigma-Aldrich) or α-tubulin (T5168; Sigma-Aldrich) signal served as loading controls. The immunoblot band intensities were quantified using ImageJ software.

### RNA Sequencing (RNA-seq) Analysis

Total RNA was isolated from MDA-MB-468 and BT-20 cells stably transfected with a control shRNA or QPRT-targeting shRNA. RNA-seq libraries were prepared and sequenced on an Illumina NovaSeq 6000 System (Illumina, San Diego, CA). Raw reads were trimmed to remove adaptor contamination and low-quality reads. Expression levels of the annotated genes were estimated using fragments per kilobase of transcript sequence per millions of base pairs (FPKM). RNA-seq data are available at the Gene Expression Omnibus (GEO) repository (https://www.ncbi.nlm.nih.gov/geo/), with GEO accession number GSE151521.

Hierarchical cluster analysis was performed as described (22). Differential gene expression calculations were done in DESeq2. GSEA was used to identify significant pathways associated with QPRT silencing.

### MMTV-PyVT Transgenic Mice

All animal experiments (MMH-AS-108-22) were conducted according to the guidelines established by the institutional animal care and use committee of MacKay Memorial Hospital. Male FVB/N-Tg(MMTV-PyVT)634Mul/J were randomly bred with wild-type C57BL/6J females (BioLASCO, Taipei, Taiwan) to obtain female mice heterozygous for the expression of the Polyoma Virus middle T antigen. Hemizygous MMTV-PyVT mice develop spontaneous mammary tumors that closely resemble the progression and morphology of human breast cancer (23). Mammary tumor formation was monitored by palpation twice a week. Upon the formation of palpable tumors, the mice were further observed for 3–4 weeks for tumor progression (24). Normal mammary gland tissue samples were obtained from wild-type female mice. Proteins extracted from mammary tumors and normal mammary tissues were subjected to western blot analysis. Anti-mouse Qprt antibody was purchased from Biorbyt, Cambridge, UK. Murine liver tissues were used as positive controls.

### Statistical Analysis

Data were expressed as mean ± SD. Statistical analyses were performed using Prism 8.3.0 (GraphPad, San Diego, CA). Comparisons of the subgroups were performed by an unpaired t-test or Jonckheere-Terpstra trend test. A two‐sided *P*‐value < 0.05 was considered statistically significant.

## Results

### Clinical Significance of QPRT Expression in Breast Cancer

**Figure 1:**
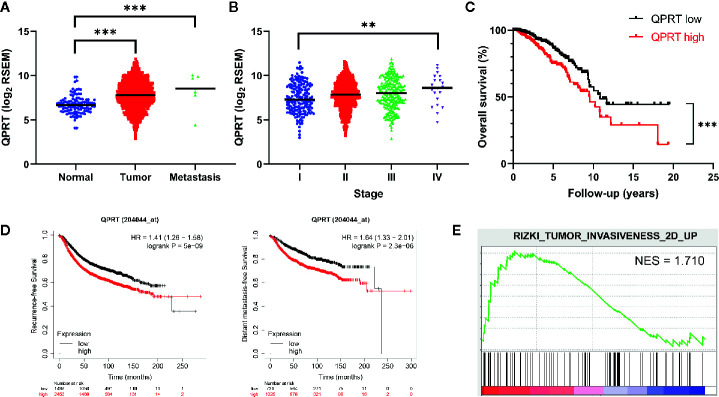
Bioinformatic analysis of the clinical significance of quinolinate phosphoribosyltransferase (QPRT) expression in breast cancer. **(A)** Expression of QPRT quantified by RNA-Seq by Expectation Maximization (RSEM) from The Cancer Genome Atlas (TCGA) breast cancer (BRCA) dataset. Significance was calculated using an unpaired t-test. ***P < 0.001. Horizontal lines represent median values. **(B)** Expression of QPRT from TCGA BRCA dataset. Significance was calculated using the Jonckheere-Terpstra trend test (n = 1071). **P _for trend_ < 0.01. Horizontal lines represent median values. **(C)** Overall survival illustrated for patients from TCGA BRCA dataset. Significance was calculated using the log-rank test. ***P < 0.001. **(D)** Recurrence-free survival and distant metastasis-free survival data from the KM Plotter. **(E)** Gene set enrichment analysis (GSEA) of differential QPRT expression using TCGA BRCA dataset NES, normalized enrichment score.

To explore the potential significance of QPRT expression in breast cancer, we set out to analyze TCGA transcriptome data. As shown in **Figure 1A**, primary breast tumors and metastatic lesions had significantly higher QPRT expression levels than normal breast tissues. Among breast cancer samples with available staging information (n = 1,071), a positive correlation between QPRT expression levels and disease stage was observed (**Figure 1B**). Divided by the median split, breast cancer patients with high QPRT expression had significantly shorter overall survival than those with low QPRT expression (*P* < 0.001, **Figure 1C**). Consistently, data from the KM Plotter indicated that breast cancer patients with high QPRT expression had significantly shorter recurrence-free and distant metastasis-free survival (**Figure 1D**). Taken together, these data indicate that higher QPRT expression may represent a negative prognostic factor in breast cancer.

GSEA was performed to identify possible alterations in association with differential QPRT expression. Interestingly, tumor invasiveness was associated with higher QPRT expression in breast cancer (**Figure 1E**).

### QPRT Protein Expression in Human and Murine Breast Neoplasms

**Figure 2:**
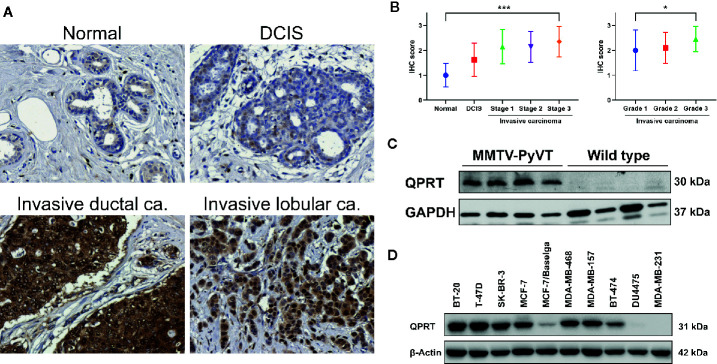
Expression of quinolinate phosphoribosyltransferase (QPRT) in human and murine breast neoplasms. **(A)** Representative QPRT immunostaining of normal breast tissue, ductal carcinoma *in situ* (DCIS), and invasive ductal/lobular carcinoma in female patients. Original magnification, 200X. **(B)** Error bar plots showing means and standard deviations of immunohistochemical (IHC) scores. Significance was calculated using the Jonckheere-Terpstra trend test (normal n = 10, DCIS n = 20, invasive cancer n = 94). *P _for trend_ < 0.05; ***P _for trend_ < 0.001. **(C)** Western blot analysis of QPRT protein expression in mammary tumors and normal mammary tissues from MMTV-PyVT and wild-type mice, respectively. **(D)** Protein expression of QPRT in a panel of breast cancer cell lines.

We next performed IHC staining in clinical breast samples. As shown in **Figure 2A**, normal breast tissue or DCIS generally showed weak QPRT immunoreactivity. Invasive ductal or lobular carcinoma exhibited moderate to strong cytoplasmic staining for QPRT. The IHC scores significantly increased from normal breast tissue (n = 10) and DCIS (n = 20) to invasive carcinoma (n = 94) of different disease stages (**Figure 2B**). A total of 76 samples of invasive carcinoma had available information of tumor grade. Among them, the IHC scores were positively correlated with higher tumor grade. Taken together, the results suggest that the QPRT protein expression in breast neoplasms was associated with the aggressiveness of breast cancer.

QPRT overexpression in breast cancer was further validated in spontaneous mammary tumors from MMTV-PyVT transgenic mice. While normal mammary gland tissues from wild-type mice exhibited virtually undetectable QPRT expression, mammary tumors of MMTV-PyVT mice had relatively abundant QPRT protein expression (**Figure 2C**).

We next screened the QPRT expression in a panel of breast cancer cell lines in our laboratory. While breast cancer cell lines had variable QPRT expression, DU4475 and MDA-MB-231 cells were negative for QPRT expression (**Figure 2D**). Accordingly, triple-negative cancer cell line MDA-MB-231 was used for gain-of-function assays, and two triple-negative cancer cell lines MDA-MB-468 and BT-20 were used for loss-of-function assays in the subsequent experiments.

### QPRT Depletion Suppressed the Migratory and Invasive Capacity

**Figure 3:**
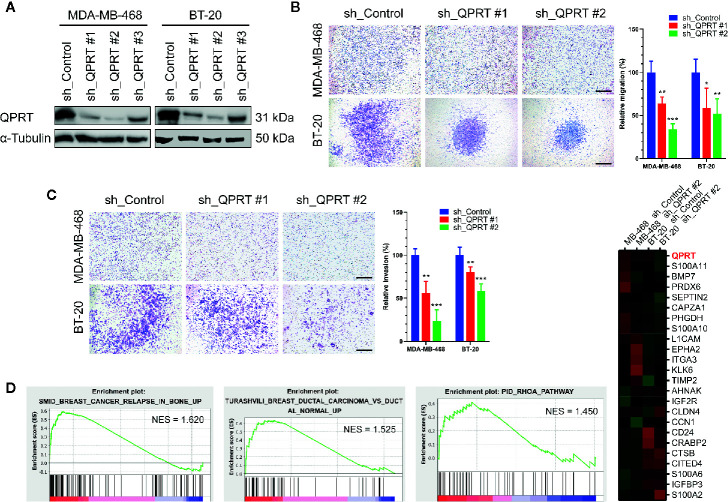
Effects of quinolinate phosphoribosyltransferase (QPRT) silencing in breast cancer cells. **(A)** Protein expression of QPRT in MDA-MB-468 and BT-20 breast cancer cells stably transfected with a control shRNA or QPRT-targeting shRNA. **(B)** Migration and **(C)** invasion determined by the Transwell assay in breast cancer cells transfected with a control shRNA or QPRT-targeting shRNA. Significance was calculated using an unpaired t-test (n = 6). *P < 0.05, **P < 0.01, ***P < 0.001. Data represent means ± standard deviations. Scale bars, 500 μm. **(D)** Heatmap of selected differentially expressed genes analyzed by RNA-seq in MDA-MB-468 and BT-20 cells transfected with a control shRNA or QPRT-targeting shRNA. Significantly enriched gene sets generated by Gene set enrichment analysis (GSEA) are shown. NES, normalized enrichment score.

Gene silencing by lentiviral shRNA transduction remarkably reduced the QPRT expression in MDA-MB-468 and BT-20 cells (**Figure 3A**). QPRT depletion did not have adverse effects on cell viability or growth in breast cancer cells (**Figure S1**). Nonetheless, the migratory and invasive capacity were significantly suppressed by QPRT knockdown (**Figures 3B, C**).

To identify differentially expressed genes in association with QPRT silencing, RNA-seq analysis was performed in MDA-MB-468 and BT-20 cells transfected with a control shRNA or QPRT-targeting shRNA. A heatmap of selected differentially expressed genes was shown in **Figure 3D**. The GSEA further suggested enrichment for gene sets associated with breast cancer bone relapse and RhoA pathway in breast cancer cells with higher QPRT expression.

### Ectopic QPRT Expression Increased the Migratory and Invasive Capacity

**Figure 4:**
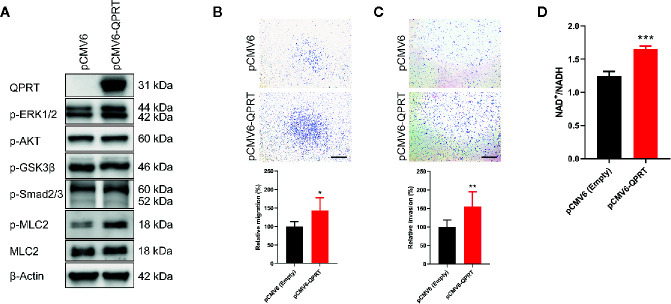
Effects of ectopic quinolinate phosphoribosyltransferase (QPRT) expression in breast cancer cells. **(A)** Protein expression of QPRT and relevant phosphorylated molecules in MDA-MB-231 cells transfected with pCMV6-empty vector or pCMV6-QPRT. **(B)** Migration and **(C)** invasion determined by the Transwell assay in MDA-MB-231 cells transfected with pCMV6-empty vector or pCMV6-QPRT. Significance was calculated using an unpaired t-test (n = 6). *P < 0.05, **P < 0.01. Data represent means ± standard deviations. Scale bars, 500 μm. **(D)** The ratio of intracellular NAD^+^ to NADH levels in MDA-MB-231 cells transfected with pCMV6-empty vector or pCMV6-QPRT. Significance was calculated using an unpaired t-test (n = 3). ***P < 0.001. Data represent means ± standard deviations.

MDA-MB-231 cells showed undetectable QPRT expression and were transfected with pCMV6-QPRT. Abundant QPRT protein expression following transfection was confirmed by Western blot (**Figure 4A**). In malignant glioma cells, QPRT expression prevented apoptosis and increased resistance to oxidative stress induced by chemoradiotherapy (12). To test the hypothesis that QPRT overexpression may exert anti-apoptotic effects in breast cancer cells, MDA-MB-231 cells transfected with pCMV6-empty vector or pCMV6-QPRT were treated with increasing doses of cisplatin. Nonetheless, we found no augmentation of cell viability in QPRT-overexpressing cells (**Figure S2**).

Following QPRT overexpression, MDA-MB-231 cells had significantly increased migratory and invasive capacity (**Figures 4B, C**). It is consistent with the results of loss-of-function assays in MDA-MB-468 and BT-20 cells. As expected, the NAD^+^/NADH ratio was increased in MDA-MB-231 cells transfected with pCMV6-QPRT (**Figure 4D**).

### QPRT Overexpression Increased the Phosphorylation of Myosin Light Chain

We next asked whether canonical oncogenic signaling pathways are involved in the QPRT-mediated increase in cellular migration and invasion. Following QPRT overexpression, the phosphorylation of ERK1/2 and myosin light chain was increased, while there was no alteration in the phosphorylation of AKT, GSK3β, and Smad2/3 (**Figure 4A**). Phosphorylation of the myosin light chain is a prime regulatory event of the contractile mechanism of stress fibers (25). Considering that the involvement of the RhoA pathway was suggested by our GSEA enrichment results, we hypothesized that the phosphorylation of myosin light chain might be the main effector of QPRT-associated invasiveness.

**Figure 5:**
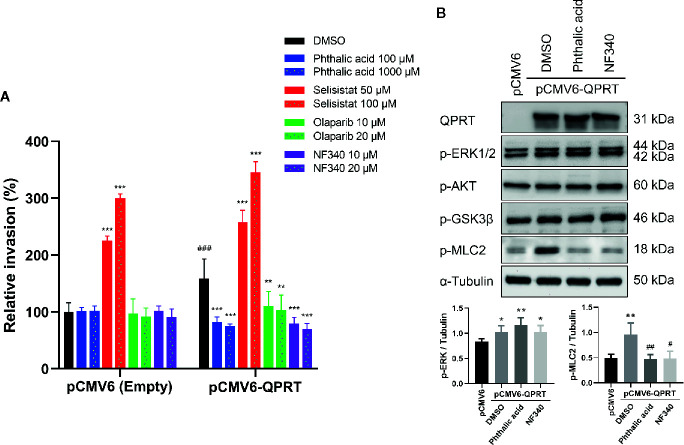
Effects of inhibitors on quinolinate phosphoribosyltransferase (QPRT)-induced invasiveness in breast cancer cells. **(A)** MDA-MB-231 cells transfected with pCMV6-empty vector or pCMV6-QPRT were treated with vehicle control (dimethyl sulfoxide; DMSO), QPRT inhibitor (phthalic acid), SIRT1 inhibitor (selisistat), PARP inhibitor (olaparib), or P2Y_11_ antagonist (NF340) for 24 h. Cell invasive ability was determined by the Transwell assay. Significance was calculated using an unpaired t-test (n = 4). **P < 0.01, ***P < 0.001 compared to DMSO control. ^###^ *P* < 0.001 compared to pCMV6-empty vector. Data represent means ± standard deviations. **(B)** Protein expression of QPRT and relevant phosphorylated molecules in MDA-MB-231 cells transfected with pCMV6-empty vector or pCMV6-QPRT and treated with vehicle control (DMSO), QPRT inhibitor (phthalic acid 100 μM), or P2Y_11_ antagonist (NF340 20 μM) for 24 h. Significance was calculated using an unpaired t-test (n = 4). *P < 0.05, **P < 0.01 compared to pCMV6-empty vector. ^#^ *P* < 0.05, ^##^ *P* < 0.01 compared to DMSO control. Data represent means ± standard deviations.

Sirtuins and poly(ADP-ribose) polymerases (PARPs) are important NAD^+^-consuming enzymes and play important roles in breast cancer biology (10). Furthermore, NAD^+^ can act as a ligand for the P2Y_11_ purinoreceptor (26). To further delineate the association between QPRT and the RhoA pathway, we used QPRT inhibitor (phthalic acid), SIRT1 inhibitor (selisistat), PARP inhibitor (olaparib), and P2Y_11_ antagonist (NF340) to examine the reversibility of the QPRT-enhanced invasiveness. At concentrations of no significant impact on the invasiveness in control cells, phthalic acid effectively reversed the augmented invasiveness in association with QPRT overexpression (**Figure 5A**). Paradoxically, selisistat heightened the invasiveness in MDA-MB-231 cells transfected with pCMV6-empty vector or pCMV6-QPRT. Olaparib and NF340 were able to reverse the QPRT-enhanced invasiveness, while the latter showed a better efficacy.

Subsequently, we evaluated the effects of treatment with phthalic acid or NF340 in QPRT-overexpressing MDA-MB-231 cells. Either treatment did not alter the phosphorylation of ERK1/2, AKT, and GSK3β. Nonetheless, both agents attenuated the phosphorylation of myosin light chain induced by ectopic QPRT expression (**Figure 5B**).

### QPRT Involved Both Rho GTPase and Phospholipase C (PLC) Pathways

**Figure 6:**
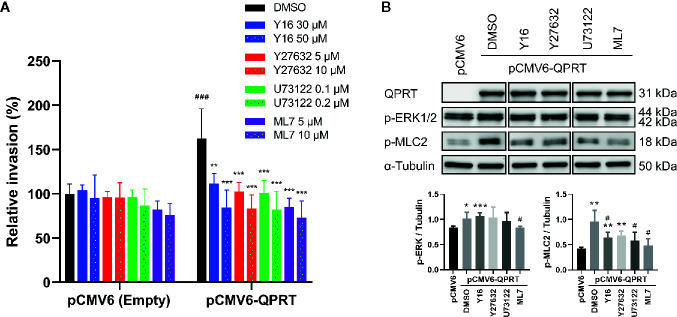
Effects of inhibitors on quinolinate phosphoribosyltransferase (QPRT)-induced invasiveness in breast cancer cells. **(A)** MDA-MB-231 cells transfected with pCMV6-empty vector or pCMV6-QPRT were treated with vehicle control (dimethyl sulfoxide; DMSO), Rho inhibitor (Y16), ROCK inhibitor (Y27632), PLC inhibitor (U73122), or MLCK inhibitor (ML7) for 24 h. Cell invasive ability was determined by the Transwell assay. Significance was calculated using an unpaired t-test (n = 4). **P < 0.01, ***P < 0.001 compared to DMSO control. ^###^ *P* < 0.001 compared to pCMV6-empty vector. Data represent means ± standard deviations. **(B)** Protein expression of QPRT and relevant phosphorylated molecules in MDA-MB-231 cells transfected with pCMV6-empty vector or pCMV6-QPRT and treated with vehicle control (DMSO), Rho inhibitor (Y16 50 μM), ROCK inhibitor (Y27632 10 μM), PLC inhibitor (U73122 0.2 μM), or MLCK inhibitor (ML7 10 μM) for 24 h. Significance was calculated using an unpaired t-test (n = 4). *P < 0.05, **P < 0.01, ***P < 0.001 compared to pCMV6-empty vector. ^#^ *P* < 0.05 compared to DMSO control. Data represent means ± standard deviations.

G-protein-coupled P2Y_11_ receptors activate PLC and stimulate inositol trisphosphate generation and calcium mobilization (27). Subsequently, Ca^2+^/calmodulin-dependent myosin light chain kinase (MLCK) is activated, resulting in phosphorylation of myosin light chain and cell contraction. Furthermore, Gαq/11 could enhance p63RhoGEF-induced RhoA activation by direct protein-protein interaction (28). RhoA and its effector, Rho kinase (ROCK), modulate the phosphorylation of myosin light chain and its dephosphorylation by myosin phosphatase (29). Accordingly, we used Rho inhibitor (Y16), ROCK inhibitor (Y27632), PLC inhibitor (U73122), and MLCK inhibitor (ML7) to assess the reversibility of the QPRT-enhanced invasiveness. At concentrations of no significant impact on the invasiveness in control cells, all inhibitors could effectively reverse the QPRT-enhanced invasiveness (**Figure 6A**).

Treatment with inhibitors of Rho, ROCK, PLC, or MLCK did not affect the QPRT expression. While the phosphorylation of ERK1/2 was reduced by the MLCK inhibitor, the most consistent finding was that the QPRT overexpression-induced phosphorylation of myosin light chain was decreased by treatment with either Rho, ROCK, PLC, or MLCK inhibitor (**Figure 6B**). Taken together, these results indicate that the Rho-ROCK and PLC-MLCK pathways are both involved in the phosphorylation of myosin light chain as a downstream of the P2Y_11_ activation.

## Discussion

In this study, we provided strong evidence supporting the association between QPRT upregulation and tumor progression in breast cancer. First, QPRT expression was positively associated with disease stage and tumor grade, whereas normal breast tissue exhibited low or undetectable QPRT expression. Second, spontaneous mammary tumors from MMTV-PyVT transgenic mice showed a similar trend of QPRT overexpression. Third, QPRT depletion significantly hindered the invasiveness of breast cancer cells.

NAD^+^ participates in various cellular physiologic processes including glycolysis and oxidative phosphorylation. In mammalian cells, NAD^+^ can be synthesized from nicotinic acid using the Preiss-Handler pathway, synthesized from tryptophan *via* the *de novo* pathway, or generated from nicotinamide or nicotinamide riboside *via* the salvage pathway. The source of NAD^+^ generation appears to be tissue and cellular context-dependent (30). Targeting NAMPT of the salvage pathway has received much attention in recent years. Nonetheless, the metabolic plasticity of cancer cells generally leads to acquired resistance to NAMPT inhibition, and increased expression or activity of QPRT is a novel resistance mechanism (31, 32). It, therefore, highlights the need to further investigate the role of QPRT in cancer biology.

The expression of QPRT in malignant neoplasms was evaluated in only a few tumor types. Increased QPRT expression was observed in glioblastomas and follicular thyroid carcinomas (12, 33). Conversely, QPRT expression was decreased in renal cell carcinoma in comparison to normal kidney tissue (34). Furthermore, QPRT expression was decreased in metastatic melanoma after the acquisition of resistance to BRAF inhibitors (35). In the present study, upregulation of QPRT expression was validated in clinical samples and spontaneous murine tumors.

Increased QPRT expression would enhance the synthesis of nicotinic acid mononucleotide and in turn NAD^+^. A reduction in intracellular NAD^+^ in breast cancer cells may induce apoptosis and suppress cell survival (36). On the contrary, nonlethal reduction of NAD^+^ may render tumor cells more aggressive and increase metastasis (37). Although QPRT seems to possess anti-apoptotic properties in some tumor types (12, 38), we did not find any difference in cell growth or viability in breast cancer cells following QPRT depletion or ectopic QPRT expression. At the time of this manuscript preparation, Yue et al. reported that knocking down QPRT in MCF-7 and T47D cells increased the apoptosis rate, and QPRT overexpression marginally decreased apoptosis (39). The basis for such a difference is unclear, but our group and Yue et al. both showed that QPRT positively participates in regulating the migratory and invasive capacity of breast cancer cells.

We further examined whether the invasion-promoting effect of QPRT was mediated by NAD^+^-consuming enzymes. Sirtuins are a family of histone deacetylases that require NAD^+^ as a substrate for SIRT-mediated deacetylation reactions. Sirtuins can promote or suppress breast cancer metastasis, and several sirtuin modulators (including selisistat, a SIRT1 inhibitor) have been investigated in clinical trials (40). In breast cancer cells, SIRT1 depletion induced an epithelial shift and inhibited cell invasion (41). Nonetheless, we found that treatment with selisistat in QPRT-overexpressing cells resulted in an uprise of cell invasion. PARPs are also NAD^+^-consuming enzymes, and poly(ADP)-ribosylation is a unique post-translational modification affecting various protein functions. The PARP inhibitor olaparib has been approved to treat patients whose cancer is positive for homologous recombination deficiency. MDA-MB-231 is a BRCA wild-type cell line with BRCA1 allelic loss and shows normal BRCA1 transcript levels (42). Although olaparib could partially reverse the QPRT-enhanced invasiveness, we were focusing on purinergic signaling as the potential link.

Nucleotides are prometastatic factors favoring tumor cell migration and tissue colonization, and purinergic receptors play an important role (43, 44). Given that NAD^+^ is a P2Y_11_ agonist (45), we evaluated the rescue effect of a P2Y_11_ antagonist NF340 and found that NF340 effectively reversed the QPRT-enhanced invasiveness. The result was similar to that treatment with NF340 prevented ATP-induced stimulation of cell migration in hepatocellular carcinoma cells (46). Furthermore, we showed that both Rho GTPase and PLC pathways downstream to the P2Y_11_ receptor were involved. Nonetheless, we could not exclude the possibility that additional NAD^+^-independent mechanisms are operative in the QPRT-mediated increment in cell migration and invasion.

Currently, phthalic acid, a quinolinic acid analog, is the only competitive inhibitor of QPRT (11). Treatment with phthalic acid reduced intracellular NAD^+^ levels, SIRT1 activity, and cell viability in a dose-dependent manner in human astrocytes and neurons (47). Although phthalates are widely used as plasticizing agents, phthalic acid is a germ cell mutagen (48). While newer selective QPRT inhibitors are under investigation, it would be interesting to determine the synergic or additive effects of combining QPRT and NAMPT inhibitors. Additionally, QPRT blockade might undoubtfully increase the upstream quinolinic acid, and quinolinic acid will lead to neurotoxicity. A combination with inhibitors against 3-hydroxyanthranilic acid oxygenase or other enzymes along the kynurenine pathway should be taken into consideration.

**Figure 7:**
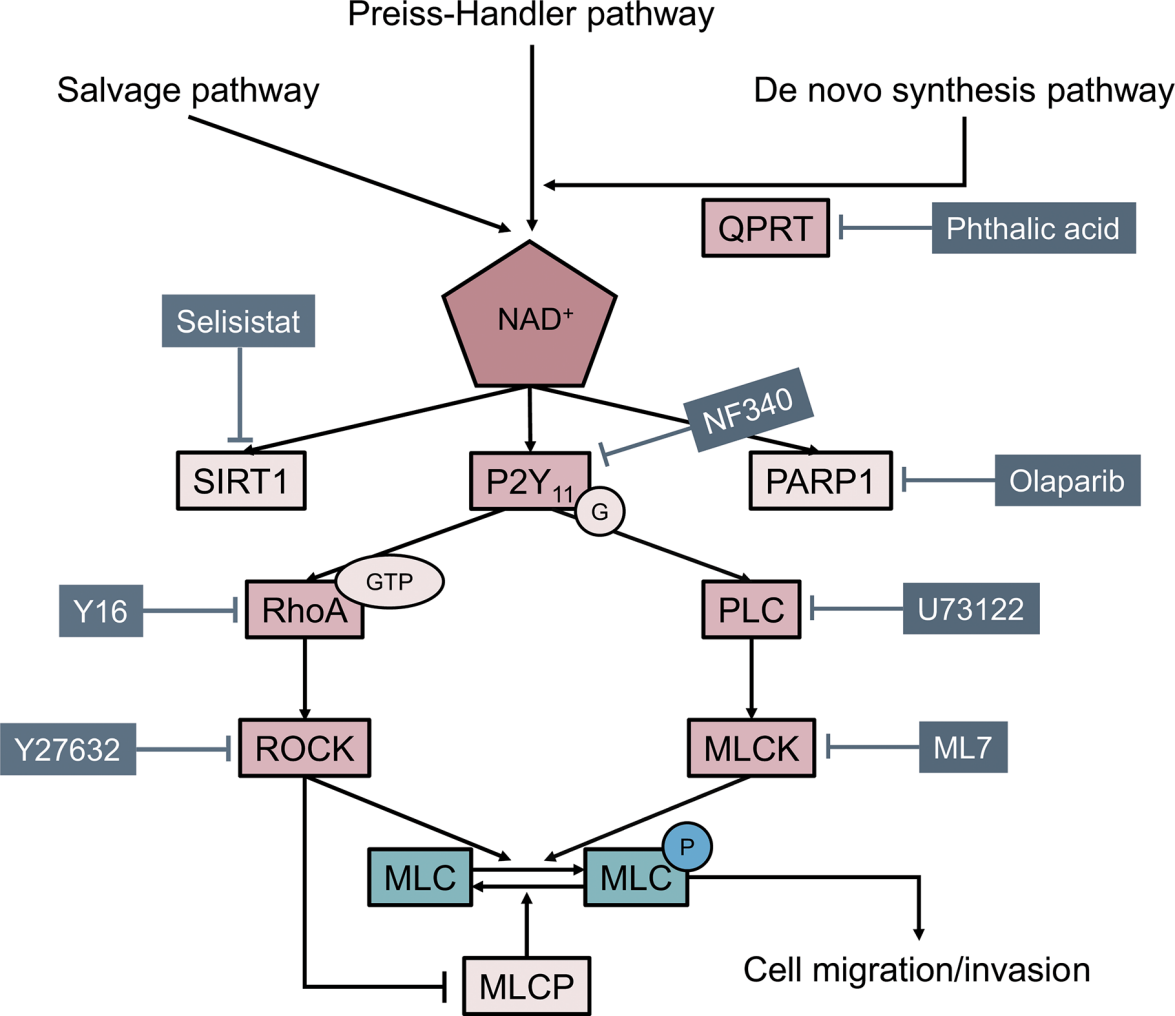
Model illustrating how quinolinate phosphoribosyltransferase (QPRT) promoting cell migration and invasion through phosphorylation of myosin light chain (MLC) in breast cancer.

In summary, the present study demonstrated a significant role of QPRT in breast cancer. QPRT promotes cell migration and invasion of breast cancer cells through, at least in part, the phosphorylation of myosin light chain *via* Rho GTPase and PLC pathways downstream of purinergic receptors (**Figure 7**). QPRT might be a potential prognostic indicator and therapeutic target in breast cancer.

## Data Availability Statement

The raw data supporting the conclusions of this article will be made available by the authors, without undue reservation.

## Ethics Statement

The animal study was reviewed and approved by Institutional Animal Care and Use Committee.

## Author Contributions

C-LL, S-NC, Y-HK, and C-HL carried out the experiment. C-LL and Y-CC wrote the manuscript with support from S-PC. M-JC helped supervised the project. Y-CC and S-PC conceived the original idea. Y-CC supervised the project. All authors contributed to the article and approved the submitted version.

## Funding

This work was supported by MacKay Memorial Hospital (grants MMH-E-107-10, MMH-E-108-10, and MMH-E-109-10).

## Conflict of Interest

The authors declare that the research was conducted in the absence of any commercial or financial relationships that could be construed as a potential conflict of interest.

## Supplementary Material

Supplementary Figure 1 Effects of quinolinate phosphoribosyltransferase (QPRT) depletion on breast cancer cell growth. In MDA-MB-468 and BT-20 cells stably transfected with a control shRNA or QPRT-targeting shRNA, cell viability was determined by the CellTiter Aqueous One Solution Cell Proliferation (MTS) Assay at 24 to 96 h. Significance was calculated using an unpaired t-test (n = 3). Data represent means ± standard deviations.

Supplementary Figure 2 Effects of ectopic quinolinate phosphoribosyltransferase (QPRT) expression on cell viability and chemotherapy sensitivity. MDA-MB-231 cells transfected with pCMV6-empty vector or pCMV6-QPRT were treated with cisplatin in serial dilutions. Cell viability was determined by the CellTiter Aqueous One Solution Cell Proliferation (MTS) Assay at 24 and 48 h. Significance was calculated using an unpaired t-test (n = 3). Data represent means ± standard deviations.

## References

[1] Heer E Harper A Escandor N Sung H McCormack V Fidler-Benaoudia MM Global burden and trends in premenopausal and postmenopausal breast cancer: a population-based study. Lancet Glob Health (2020) 8(8):e1027–37. 10.1016/S2214-109X(20)30215-1 32710860

[2] Chen Z Xu L Shi W Zeng F Zhuo R Hao X Trends of female and male breast cancer incidence at the global, regional, and national levels, 1990-2017. Breast Cancer Res Treat (2020) 180(2):481–90. 10.1007/s10549-020-05561-1 32056055

[3] Chiang CJ Lo WC Yang YW You SL Chen CJ Lai MS Incidence and survival of adult cancer patients in Taiwan, 2002-2012. J Formos Med Assoc (2016) 115(12):1076–88. 10.1016/j.jfma.2015.10.011 26786251

[4] Ho YR Ma SP Chang KY Trends in regional cancer mortality in Taiwan 1992-2014. Cancer Epidemiol (2019) 59:185–92. 10.1016/j.canep.2019.02.005 30825841

[5] Katsyuba E Romani M Hofer D Auwerx J NAD(+) homeostasis in health and disease. Nat Metab (2020) 2(1):9–31. 10.1038/s42255-019-0161-5 32694684

[6] Verdin E NAD(+) in aging, metabolism, and neurodegeneration. Science (2015) 350(6265):1208–13. 10.1126/science.aac4854 26785480

[7] Pramono AA Rather GM Herman H Lestari K Bertino JR NAD- and NADPH-Contributing Enzymes as Therapeutic Targets in Cancer: An Overview. Biomolecules (2020) 10(3):358. 10.3390/biom10030358 PMC7175141 32111066

[8] Lee YC Yang YH Su JH Chang HL Hou MF Yuan SS High visfatin expression in breast cancer tissue is associated with poor survival. Cancer Epidemiol Biomarkers Prev (2011) 20(9):1892–901. 10.1158/1055-9965.EPI-11-0399 21784959

[9] Ji C Cong R Wang Y Wang Y Zhang Q Zhou X Relationship between NAMPT/PBEF/visfatin and prognosis of patients with malignant tumors: a systematic review and meta-analysis. Ann Transl Med (2019) 7(23):785. 10.21037/atm.2019.11.32 32042801 PMC6989992

[10] Canto C Menzies KJ Auwerx J NAD(+) Metabolism and the Control of Energy Homeostasis: A Balancing Act between Mitochondria and the Nucleus. Cell Metab (2015) 22(1):31–53. 10.1016/j.cmet.2015.05.023 26118927 PMC4487780

[11] Jacobs KR Castellano-Gonzalez G Guillemin GJ Lovejoy DB Major Developments in the Design of Inhibitors along the Kynurenine Pathway. Curr Med Chem (2017) 24(23):2471–95. 10.2174/0929867324666170502123114 PMC5748880 28464785

[12] Sahm F Oezen I Opitz CA Radlwimmer B von Deimling A Ahrendt T The endogenous tryptophan metabolite and NAD+ precursor quinolinic acid confers resistance of gliomas to oxidative stress. Cancer Res (2013) 73(11):3225–34. 10.1158/0008-5472.CAN-12-3831 23548271

[13] Xu YH Deng JL Wang LP Zhang HB Tang L Huang Y Identification of Candidate Genes Associated with Breast Cancer Prognosis. DNA Cell Biol (2020) 39(7):1205–27. 10.1089/dna.2020.5482 32456464

[14] Chen IC Hsiao LP Huang IW Yu HC Yeh LC Lin CH Phosphatidylinositol-3 Kinase Inhibitors, Buparlisib and Alpelisib, Sensitize Estrogen Receptor-positive Breast Cancer Cells to Tamoxifen. Sci Rep (2017) 7(1):9842. 10.1038/s41598-017-10555-z 28852212 PMC5574981

[15] N. Cancer Genome Atlas Comprehensive molecular portraits of human breast tumours. Nature (2012) 490(7418):61–70. 10.1038/nature11412 23000897 PMC3465532

[16] Subramanian A Tamayo P Mootha VK Mukherjee S Ebert BL Gillette MA Gene set enrichment analysis: a knowledge-based approach for interpreting genome-wide expression profiles. Proc Natl Acad Sci U.S.A. (2005) 102(43):15545–50. 10.1073/pnas.0506580102 PMC1239896 16199517

[17] Gyorffy B Lanczky A Eklund AC Denkert C Budczies J Li Q An online survival analysis tool to rapidly assess the effect of 22,277 genes on breast cancer prognosis using microarray data of 1,809 patients. Breast Cancer Res Treat (2010) 123(3):725–31. 10.1007/s10549-009-0674-9 20020197

[18] Chen IC Chang YC Lu YS Chung KP Huang CS Lu TP Clinical Relevance of Liver Kinase B1(LKB1) Protein and Gene Expression in Breast Cancer. Sci Rep (2016) 6:21374. 10.1038/srep21374 26877155 PMC4753425

[19] Chang YC Lin CH Lin JC Cheng SP Chen SN Liu CL Inhibition of 3beta-Hydroxysteroid Dehydrogenase Type 1 Suppresses Interleukin-6 in Breast Cancer. J Surg Res (2019) 241:8–14. 10.1016/j.jss.2019.03.024 31004874

[20] Liu CL Chen MJ Lin JC Lin CH Huang WC Cheng SP Doxorubicin Promotes Migration and Invasion of Breast Cancer Cells through the Upregulation of the RhoA/MLC Pathway. J Breast Cancer (2019) 22(2):185–95. 10.4048/jbc.2019.22.e22 PMC6597404 31281722

[21] Chang YC Chen CK Chen MJ Lin JC Lin CH Huang WC Expression of 3beta-Hydroxysteroid Dehydrogenase Type 1 in Breast Cancer is Associated with Poor Prognosis Independent of Estrogen Receptor Status. Ann Surg Oncol (2017) 24(13):4033–41. 10.1245/s10434-017-6000-6 28744792

[22] Cheng SP Chen MJ Chien MN Lin CH Lee JJ Liu CL Overexpression of teneurin transmembrane protein 1 is a potential marker of disease progression in papillary thyroid carcinoma. Clin Exp Med (2017) 17(4):555–64. 10.1007/s10238-016-0445-y 28004221

[23] Lin EY Jones JG Li P Zhu L Whitney KD Muller WJ Progression to malignancy in the polyoma middle T oncoprotein mouse breast cancer model provides a reliable model for human diseases. Am J Pathol (2003) 163(5):2113–26. 10.1016/S0002-9440(10)63568-7 PMC1892434 14578209

[24] Chang YC Liu CL Chen MJ Hsu YW Chen SN Lin CH Local anesthetics induce apoptosis in human breast tumor cells. Anesth Analg (2014) 118(1):116–24. 10.1213/ANE.0b013e3182a94479 24247230

[25] Katoh K Kano Y Noda Y Rho-associated kinase-dependent contraction of stress fibres and the organization of focal adhesions. J R Soc Interface (2011) 8(56):305–11. 10.1098/rsif.2010.0419 PMC3030825 20826475

[26] Moreschi I Bruzzone S Nicholas RA Fruscione F Sturla L Benvenuto F Extracellular NAD+ is an agonist of the human P2Y11 purinergic receptor in human granulocytes. J Biol Chem (2006) 281(42):31419–29. 10.1074/jbc.M606625200 16926152

[27] Communi D Govaerts C Parmentier M Boeynaems JM Cloning of a human purinergic P2Y receptor coupled to phospholipase C and adenylyl cyclase. J Biol Chem (1997) 272(51):31969–73. 10.1074/jbc.272.51.31969 9405388

[28] Lutz S Freichel-Blomquist A Yang Y Rumenapp U Jakobs KH Schmidt M The guanine nucleotide exchange factor p63RhoGEF, a specific link between Gq/11-coupled receptor signaling and RhoA. J Biol Chem (2005) 280(12):11134–9. 10.1074/jbc.M411322200 15632174

[29] Amin E Dubey BN Zhang SC Gremer L Dvorsky R Moll JM Rho-kinase: regulation, (dys)function, and inhibition. Biol Chem (2013) 394(11):1399–410. 10.1515/hsz-2013-0181 PMC5538733 23950574

[30] Chowdhry S Zanca C Rajkumar U Koga T Diao Y Raviram R NAD metabolic dependency in cancer is shaped by gene amplification and enhancer remodelling. Nature (2019) 569(7757):570–5. 10.1038/s41586-019-1150-2 PMC7138021 31019297

[31] Guo J Lam LT Longenecker KL Bui MH Idler KB Glaser KB Identification of novel resistance mechanisms to NAMPT inhibition via the de novo NAD(+) biosynthesis pathway and NAMPT mutation. Biochem Biophys Res Commun (2017) 491(3):681–6. 10.1016/j.bbrc.2017.07.143 28756225

[32] Thongon N Zucal C D’Agostino VG Tebaldi T Ravera S Zamporlini F Cancer cell metabolic plasticity allows resistance to NAMPT inhibition but invariably induces dependence on LDHA. Cancer Metab (2018) 6:1. 10.1186/s40170-018-0174-7 29541451 PMC5844108

[33] Hinsch N Frank M Doring C Vorlander C Hansmann ML QPRT: a potential marker for follicular thyroid carcinoma including minimal invasive variant; a gene expression, RNA and immunohistochemical study. BMC Cancer (2009) 9:93. 10.1186/1471-2407-9-93 19321014 PMC2667537

[34] Hornigold N Dunn KR Craven RA Zougman A Trainor S Shreeve R Dysregulation at multiple points of the kynurenine pathway is a ubiquitous feature of renal cancer: implications for tumour immune evasion. Br J Cancer (2020) 123(1):13s7–147. 10.1038/s41416-020-0874-y PMC7341846 32390008

[35] Audrito V Manago A La Vecchia S Zamporlini F Vitale N Baroni G Nicotinamide Phosphoribosyltransferase (NAMPT) as a Therapeutic Target in BRAF-Mutated Metastatic Melanoma. J Natl Cancer Inst (2018) 110(3):290–303. 10.1093/jnci/djx198 29309612

[36] Bolandghamat Pour Z Nourbakhsh M Mousavizadeh K Madjd Z Ghorbanhosseini SS Abdolvahabi Z Up-regulation of miR-381 inhibits NAD+ salvage pathway and promotes apoptosis in breast cancer cells. EXCLI J (2019) 18:683–96. 10.17179/excli2019-1431 PMC6785761 31611752

[37] Santidrian AF Matsuno-Yagi A Ritland M Seo BB LeBoeuf SE Gay LJ Mitochondrial complex I activity and NAD+/NADH balance regulate breast cancer progression. J Clin Invest (2013) 123(3):1068–81. 10.1172/JCI64264 PMC3582128 23426180

[38] Ullmark T Montano G Jarvstrat L Jernmark Nilsson H Hakansson E Drott K Anti-apoptotic quinolinate phosphoribosyltransferase (QPRT) is a target gene of Wilms’ tumor gene 1 (WT1) protein in leukemic cells. Biochem Biophys Res Commun (2017) 482(4):802–7. 10.1016/j.bbrc.2016.11.114 27889611

[39] Yue Z Shusheng J Hongtao S Shu Z Lan H Qingyuan Z Silencing DSCAM-AS1 suppresses the growth and invasion of ER-positive breast cancer cells by downregulating both DCTPP1 and QPRT. Aging (Albany NY) (2020) 12(14):14754–74. 10.18632/aging.103538 PMC7425442 32716908

[40] Sinha S Sharma S Vora J Shrivastava N Emerging role of sirtuins in breast cancer metastasis and multidrug resistance: Implication for novel therapeutic strategies targeting sirtuins. Pharmacol Res (2020) 158:104880. 10.1016/j.phrs.2020.104880 32442721

[41] Shi L Tang X Qian M Liu Z Meng F Fu L A SIRT1-centered circuitry regulates breast cancer stemness and metastasis. Oncogene (2018) 37(49):6299–315. 10.1038/s41388-018-0370-5 PMC6283862 30038266

[42] Elstrodt F Hollestelle A Nagel JH Gorin M Wasielewski M van den Ouweland A BRCA1 mutation analysis of 41 human breast cancer cell lines reveals three new deleterious mutants. Cancer Res (2006) 66(1):41–5. 10.1158/0008-5472.CAN-05-2853 16397213

[43] Ferrari D Malavasi F Antonioli L A Purinergic Trail for Metastases. Trends Pharmacol Sci (2017) 38(3):277–90. 10.1016/j.tips.2016.11.010 27989503

[44] Bellefeuille SD Molle CM Gendron FP Reviewing the role of P2Y receptors in specific gastrointestinal cancers. Purinergic Signal (2019) 15(4):451–63. 10.1007/s11302-019-09678-x PMC6923304 31478181

[45] Kennedy C P2Y11 Receptors: Properties, Distribution and Functions. Adv Exp Med Biol (2017) 1051:107–22. 10.1007/5584_2017_89 29134605

[46] Khalid M Brisson L Tariq M Hao Y Guibon R Fromont G Carcinoma-specific expression of P2Y11 receptor and its contribution in ATP-induced purinergic signalling and cell migration in human hepatocellular carcinoma cells. Oncotarget (2017) 8(23):37278–90. 10.18632/oncotarget.16191 PMC5514908 28418839

[47] Braidy N Guillemin GJ Grant R Effects of Kynurenine Pathway Inhibition on NAD Metabolism and Cell Viability in Human Primary Astrocytes and Neurons. Int J Tryptophan Res (2011) 4:29–37. 10.4137/IJTR.S7052 22084601 PMC3195218

[48] Jha AM Singh AC Bharti M Germ cell mutagenicity of phthalic acid in mice. Mutat Res (1998) 422(2):207–12. 10.1016/s0027-5107(98)00151-1 9838120

