# Supplementary figures and images for: Quinolinate Phosphoribosyltransferase Promotes Invasiveness of Breast Cancer Through Myosin Light Chain Phosphorylation
Source: Front Endocrinol (Lausanne). 2021 Feb 4;11:621944. doi: 10.3389/fendo.2020.621944 (PMC7890081; doi:10.3389/fendo.2020.621944)

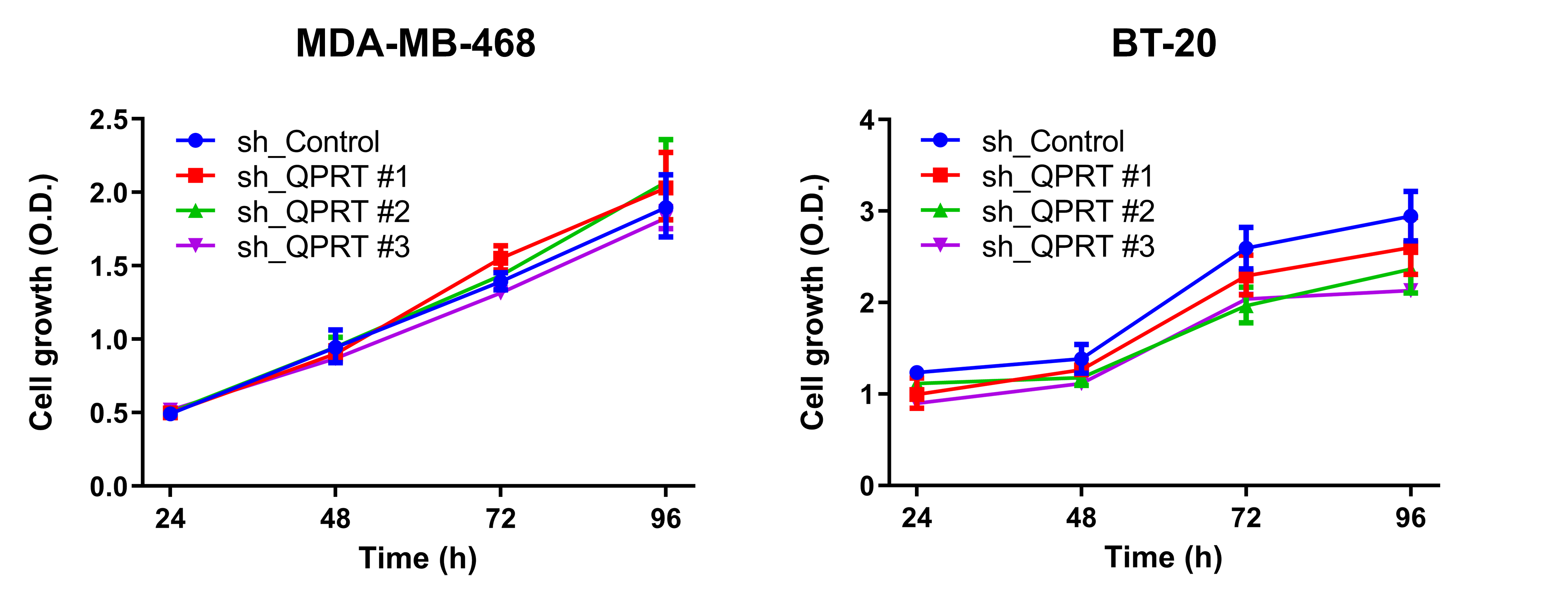

Supplement: Supplementary Figure 1 — Effects of quinolinate phosphoribosyltransferase (QPRT) depletion on breast cancer cell growth. In MDA-MB-468 and BT-20 cells stably transfected with a control shRNA or QPRT-targeting shRNA, cell viability was determined by the CellTiter Aqueous One Solution Cell Proliferation (MTS) Assay at 24 to 96 h. Significance was calculated using an unpaired t-test (n = 3). Data represent means ± standard deviations. [file Image_1.tif]

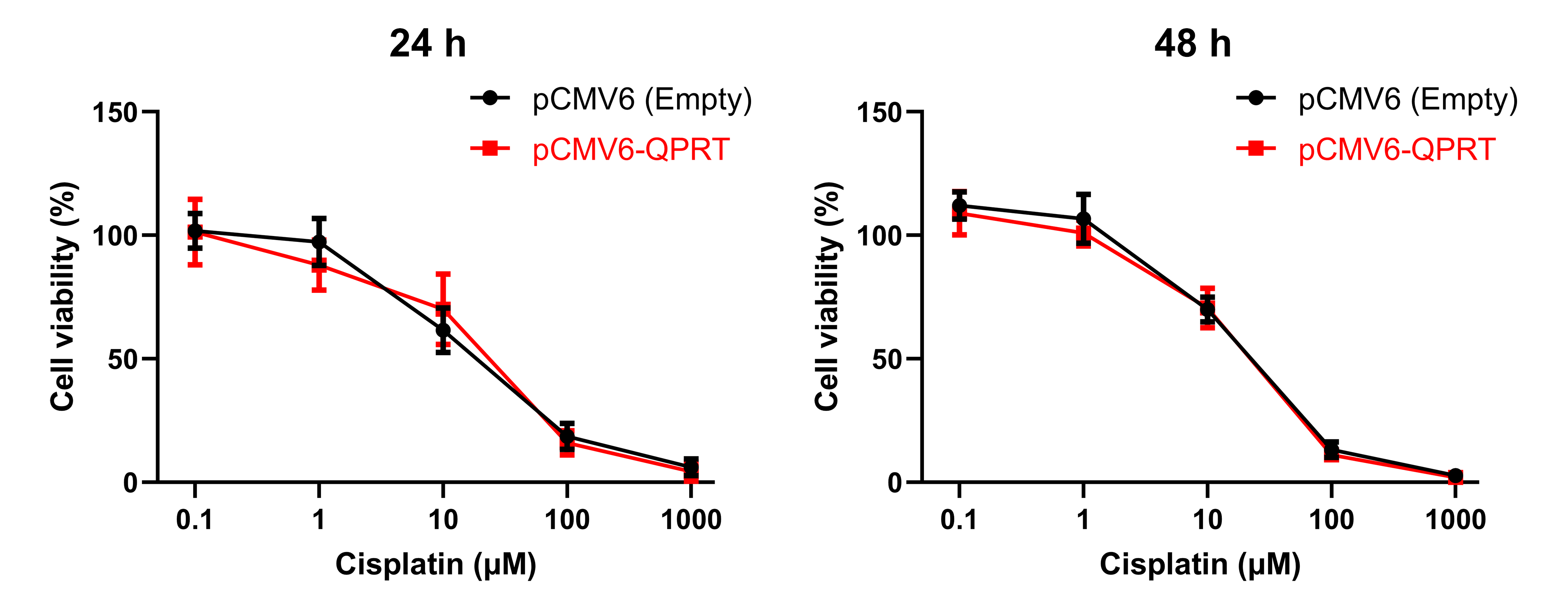

Supplement: Supplementary Figure 2 — Effects of ectopic quinolinate phosphoribosyltransferase (QPRT) expression on cell viability and chemotherapy sensitivity. MDA-MB-231 cells transfected with pCMV6-empty vector or pCMV6-QPRT were treated with cisplatin in serial dilutions. Cell viability was determined by the CellTiter Aqueous One Solution Cell Proliferation (MTS) Assay at 24 and 48 h. Significance was calculated using an unpaired t-test (n = 3). Data represent means ± standard deviations. [file Image_2.tif]
